# Rater Behavior as an Enactment Condition in Nursing Performance Appraisal: A Critical Review

**DOI:** 10.1155/jonm/4829440

**Published:** 2026-11-15

**Authors:** Ehab Tumeh, Nehad Fekri, Magda Almolla

**Affiliations:** ^1^ Department of Nursing Ibn Sina College for Health Professions Nablus University for Vocational and Technical Education Nablus State of Palestine; ^2^ Faculty of Nursing Cairo University Cairo Egypt cu.edu.eg

**Keywords:** enactment condition, nurses, nursing management, organizational justice, performance appraisal, psychometric properties, rating quality

## Abstract

**Aim(s):**

To critically synthesise and evaluate evidence on rater behavior, score information and fairness perceptions in nursing performance appraisal and to propose a limited, testable conceptual framework that reframes rater behavior as an enactment condition of performance measurement, that is, a contextual condition of score production arising from how raters apply an instrument in practice.

**Background:**

Performance appraisal scores are produced through interaction between assessment instruments, raters and organizational context. Nursing research has examined rater behavior, measurement properties and fairness largely as separate concerns. The relationship between rating quality and broader psychometric properties (including reliability, validity and fairness) remains insufficiently integrated.

**Evaluation:**

A critical narrative review was conducted. PubMed/MEDLINE, CINAHL, PsycINFO and Scopus were searched on 6 September 2026, supported by reference‐list checking and citation tracking. Fifty‐one sources were included and synthesised according to nursing relevance, methodological characteristics and contribution to the proposed mechanisms. A two‐dimensional evidence classification framework distinguished direct acute‐care nursing evidence from nursing evidence outside acute care and from general performance‐assessment research. Reporting quality was guided by the Scale for the Quality Assessment of Narrative Review Articles (SANRA), applied as an author self‐assessment.

**Key Issues:**

Large‐scale evidence from nurse‐anesthetist evaluations demonstrates that systematic rating behavior can reduce score information and compromise relative ranking. Nursing fairness studies establish associations between perceived appraisal fairness and work outcomes but do not establish that measurement properties cause fairness perceptions. Psychometric studies demonstrate measurement properties without adequately examining rating behavior in practice. Reliability, validity and fairness are related but nonequivalent; restricted score distributions should not automatically be interpreted as evidence of poor validity without considering the intended measurement purpose.

**Conclusion(s):**

Rater behavior should be considered an enactment condition of performance measurement. Three propositions are advanced: (1) rater behavior systematically alters score information, a proposition with direct empirical support; (2) the consequences of rater behavior depend on whether the appraisal system requires differentiation, a primarily theoretical and methodological proposition; and (3) the relationship between score properties and perceived fairness depends on how nurses interpret the credibility and legitimacy of score differentiation, a proposition requiring direct empirical testing in nursing contexts.

**Implications for Nursing Management:**

Appraisal‐system evaluation should consider rater behavior alongside instrument properties. Research should simultaneously examine rater behavior, score distributions, measurement properties and fairness perceptions. Recommendations grounded in existing evidence should be distinguished from proposals requiring evaluation in nursing settings.

## 1. Introduction

Performance appraisal in nursing is simultaneously a measurement process, a social judgment and an organizational decision [1]. In acute care settings, appraisal scores inform competency verification, promotion, retention and professional development. Administrative decisions require defensible precision and differentiation among nurses; developmental feedback requires nuanced, supportive information that motivates improvement. When a single system must serve both purposes, tensions emerge between measurement accuracy, perceived fairness and practical utility [1].

The literature on rating quality has developed multiple approaches to defining and evaluating the adequacy of performance ratings. Reliability and agreement approaches use internal consistency and inter‐rater indices to judge whether ratings are consistent, although such indices alone cannot show that ratings are accurate [2, 3]. Measurement‐model approaches, including Generalizability Theory (G‐theory) and many‐facet Rasch measurement, partition score variance and separate rater effects from genuine performance differences [4–7]. Rater‐effect approaches document and quantify leniency, severity, halo and idiosyncratic rater contributions to scores [8–11]. Measurement‐property frameworks, such as the COnsensus‐based Standards for the selection of health Measurement INstruments (COSMIN), evaluate the quality of instruments rather than of raters [12–15]. Each approach addresses distinct aspects of rating quality and connects to broader psychometric concerns regarding reliability, validity and fairness. Understanding these connections is essential for evaluating how well performance appraisal systems function in nursing contexts.

The rater occupies a central position in this system. A performance rating is not produced by an instrument alone; it is produced by a rater using an instrument. The rater observes behavior, recalls it, integrates it with prior knowledge, evaluates it against standards and assigns a score. At each stage, rater cognition, motivation and context influence the output. Yet conventional approaches to assessment validity often treat rater behavior as a source of error surrounding an otherwise adequate instrument, rather than as a constitutive part of the measurement process itself [16, 17].

The broader literature has long recognised that appraisal is a social, cognitive, contextual and relational process [1, 18]. Erdogan [19] explicitly identified rater behavior as both a cause and a consequence of appraisal justice. Research on conscious rating distortion has examined situations where managers rate for motives other than accuracy [20, 21]. What remains underdeveloped is an integrated, critical account of how rater behavior, the informational properties of scores and fairness perceptions function together and which of these relationships are empirically established versus theoretically proposed.

Contemporary psychometric frameworks conceptualise validity not as a property of an assessment instrument itself, but as a property of score interpretations for specific uses [17, 22]. The Standards for Educational and Psychological Testing [22], published jointly by the American Educational Research Association (AERA), the American Psychological Association and the National Council on Measurement in Education, articulate that validity evidence must be accumulated for each intended interpretation and use of scores. Similarly, fairness in assessment is conceptualised as a multifaceted concern encompassing equitable treatment, absence of construct‐irrelevant variance and appropriate score interpretation for diverse populations [22]. These frameworks provide essential context for understanding why rater behavior, which directly shapes the scores produced, is consequential for validity and fairness, not merely for reliability.

Three research traditions have each accumulated substantial evidence relevant to performance appraisal in nursing, yet they have developed largely in isolation. Organizational justice research has examined the antecedents and consequences of fairness perceptions in nursing contexts [23–29], but rarely addresses rater cognition or measurement precision. Rater behavior research has documented leniency, halo, central tendency and idiosyncratic rater effects across performance evaluation contexts [4, 5, 8–11, 30–32], but seldom connects to how nurses perceive fairness or to the psychometric properties of the instruments being used. Psychometric validation research has advanced the development and cross‐cultural adaptation of nursing performance instruments [33–36], but validation samples may not reflect real‐world rating behavior.

The gap is not merely that this literature is insufficiently integrated. Existing approaches often conceptualise rater behavior, perceived fairness and measurement quality as analytically separable components of performance assessment. The reviewed evidence provides a basis for examining whether these components are dynamically interdependent: appraisal‐system design shapes rater motivation and behavior; rater behavior changes the informational properties of ratings; and those rating properties may influence how the assessment process is interpreted as fair or unfair. The recent systematic review by Sucipto et al. [37] identified persistent challenges in nursing appraisal instruments but did not develop an integrated framework linking fairness perceptions, rater behavior and measurement consequences.

The proposed framework differs from existing approaches in three respects. First, unlike rater‐effect frameworks that quantify rater variance as statistical error, it treats rater behavior as a constitutive condition of score production, an ‘enactment condition’ rather than a nuisance parameter. Second, unlike justice research that examines fairness perceptions without modeling measurement properties, it explicitly links the informational properties of scores to fairness perceptions as a testable, contingent relationship. Third, unlike the Sucipto et al. [37] systematic review, which focused on instrument validity and reliability, it integrates rater behavior, score information and fairness into a single, limited, testable model. The framework is therefore not a new measurement property; it is a conceptual synthesis that identifies which relationships are empirically established and which require direct testing.

This review is positioned within nursing management because the nursing context creates specific conditions under which rater behavior becomes consequential. In acute care settings, performance is multidimensional, observation is often episodic, multiple raters may differ in opportunity and perspective, performance is team‐dependent and ratings carry both developmental and administrative consequences [38, 39]. Nurse managers are simultaneously raters, recipients of appraisal systems and decision‐makers who use appraisal data. Understanding how their rating behavior shapes the information available for decisions is therefore a core concern for nursing management.

The aim of this review is to critically synthesise and evaluate the literature on rater behavior, score information and fairness perceptions in nursing performance appraisal and to propose a narrower, testable framework that reframes rater behavior as an enactment condition of performance measurement. Three analytical questions structure the review:

1. What methods and approaches are used to evaluate rating quality in nursing performance appraisal?
2. What does the most direct empirical evidence establish about how rater behavior alters the informational properties of performance scores in nursing?
3. What are the consequences and impacts of rater behavior, including rater effects and fairness concerns, and what framework can integrate these mechanisms?

In this review, rater behavior refers to observable or inferable patterns in how raters assign scores, including leniency, severity, central tendency, halo, uniform rating and strategic distortion. Rater effects refer to the statistical contribution of raters to observed score variation. Rater behavior can produce rater effects, but the two concepts are not interchangeable. Rating quality refers to the extent to which ratings provide accurate, reliable and useful information for their intended purpose. Enactment condition refers to a contextual condition of score production arising from how raters interpret and apply an assessment instrument in practice. The term is used here as a conceptual synthesis rather than as a new measurement property. It emphasises that score meaning depends partly on how the instrument is enacted in practice, without implying that raters are the sole determinant of validity.

The following terms are used consistently throughout and are not treated as interchangeable. Reliability refers to the consistency of scores across raters, occasions, or items. Validity refers to the degree to which evidence and theory support the interpretation of scores for their intended use [17, 22]. Score differentiation refers to the extent to which scores distinguish among ratees. Score information refers to the amount of discriminating information carried by a set of scores; information loss occurs when scores become uniform or insufficiently variable to distinguish ratees, as quantified by binomial entropy in the nurse‐anesthetist studies [10]. Accuracy refers to the correspondence between ratings and true performance. Fairness is used in two senses: perceived fairness (organizational justice) refers to ratees’ perceptions of the appraisal process and its outcomes [23], whereas fairness in the measurement sense refers to the absence of construct‐irrelevant variance and the equitable interpretation of scores [22]. Reliability, validity, differentiation, information, accuracy and fairness are related but nonequivalent properties.

## 2. Methods

### 2.1. Study Design

This study employed a critical narrative review design. Narrative review methodology was selected because the review aimed to integrate evidence from three distinct bodies of literature, organizational justice, rater behavior and psychometric research, that employ different methodological traditions and address different aspects of performance appraisal. A critical narrative approach permits interpretive synthesis across heterogeneous evidence types while maintaining explicit attention to the strengths and limitations of the evidence base [40, 41]. Unlike a systematic review with a single, narrow question, a critical narrative review is appropriate when the objective is to develop a conceptual framework and to distinguish empirically established relationships from theoretically proposed ones. The approach is therefore congruent with the review’s aim of proposing a testable framework rather than producing a pooled effect estimate.

### 2.2. Search Strategy

The following databases were searched: PubMed/MEDLINE, CINAHL, PsycINFO and Scopus. Three core search blocks and one contextual block were used. The first block addressed appraisal and comprised the terms ‘performance appraisal,’ ‘performance evaluation,’ ‘performance assessment’, ‘performance review’ and ‘employee evaluation’. The second block addressed nursing and comprised the terms ‘nurse’, ‘nursing’, ‘nurse manager’, and ‘staff nurse’. The third block addressed mechanism and comprised the terms ‘fairness’, ‘justice’, ‘rater bias’, ‘rater effect’, ‘leniency’, ‘severity’, ‘halo’, ‘central tendency’, ‘rater motivation’, ‘appraisal motivation’, ‘rating accuracy’, ‘reliability’, ‘validity’, ‘measurement error’ and ‘psychometric’.

A fourth, contextual block (‘acute care’ OR ‘hospital’) was applied in PubMed/MEDLINE, CINAHL and Scopus but not in PsycINFO. Minor database‐specific differences in controlled vocabulary and term lists are shown in Supporting File 2; for example, ‘employee evaluation’ was searched in PubMed/MEDLINE and CINAHL but not in PsycINFO or Scopus. The absence of the contextual block in PsycINFO may have increased sensitivity in that database but reduced specificity; conversely, the presence of the contextual block in the other three databases may have increased specificity but reduced sensitivity for studies in nonacute settings. To address the potential restrictiveness of this strategy, supporting reference‐list checking and citation tracking were conducted for seminal works on rater bias, organizational justice and nursing performance measurement. This supporting approach was designed to identify relevant studies addressing rater behavior and performance appraisal in organizational settings beyond acute care. The complete search string for each database is provided in Supporting File 2. A sensitivity search without the contextual filter was not undertaken because the review aimed to integrate mechanism‐focused evidence with direct relevance to nursing, and removing the filter would be expected to return a very large volume of general organizational literature that could not be screened and appraised at the same depth; the consequences of this decision are acknowledged in the Limitations.

Boolean operators (AND, OR) were used to combine search blocks. Truncation (∗) was applied to key terms (e.g., nurs∗, evaluat∗) to capture variant word endings; the truncated forms used in each database are shown in Supporting File 2. Date limits (2000–2026) were applied directly in the database interfaces, and seminal earlier works were identified through reference‐list checking and citation tracking. MeSH terms were used in PubMed where available.

The searches were conducted on 6 September 2026. Of 1292 records identified (1247 from databases; 45 from reference lists and citation tracking), 312 duplicates were removed, leaving 980 records screened at title and abstract level. After 812 records were excluded, 168 full texts were assessed for eligibility; 117 were subsequently excluded, yielding 51 studies included in the final synthesis. Supporting searches included reference lists of main articles and citation tracking for seminal works on rater bias, organizational justice and nursing performance measurement. Figure 1 presents the study selection flow diagram.

**FIGURE 1:**
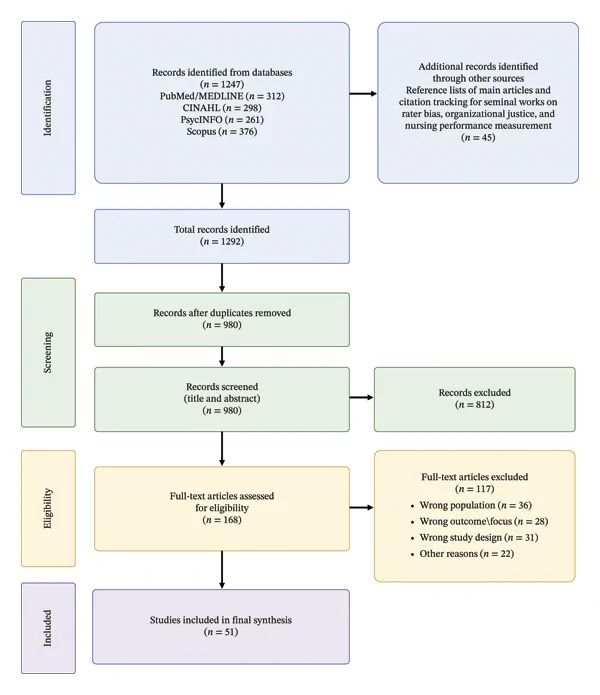
Study selection flow diagram. The diagram was created originally by the authors; no copyright permission is required. The figure shows identification (records from databases and other sources), screening (records after duplicates removed, records screened, records excluded), eligibility (full‐text articles assessed and excluded with reasons) and inclusion (studies included in the final synthesis). The arithmetic reconciles as follows: 1247 database records + 45 other records = 1292 total; 1292 − 312 duplicates = 980 screened; 980 − 812 excluded = 168 full texts assessed; 168 − 117 excluded = 51 included.

### 2.3. Eligibility Criteria

Studies were eligible for inclusion if they met the following criteria:

#### 2.3.1. Inclusion Criteria

• Addressed performance appraisal, performance evaluation, or performance assessment in nursing or healthcare organizational contexts
• Addressed rater behavior, rater effects, rating quality, measurement properties, or fairness perceptions
• Published in peer‐reviewed journals or as seminal theoretical works
• Written in English
• Published between 2000 and 2026, with seminal earlier works included as theoretical anchors

#### 2.3.2. Exclusion Criteria

• Studies focused exclusively on patient outcomes without relevance to rater behavior or appraisal processes
• Studies addressing performance appraisal in nonhealthcare settings without transferable mechanisms relevant to nursing
• Conference abstracts, editorials, or non–peer‐reviewed publications
• Studies not available in full text

Studies from general organizational contexts were included when they directly informed mechanisms of rater cognition, rating behavior, measurement error, or appraisal justice. The rationale for including non‐nursing studies was to capture theoretical and methodological advances in rating quality evaluation that have not yet been applied in nursing contexts. These studies were distinguished from nursing‐specific evidence during synthesis and were not counted among the direct acute‐care nursing evidence. Studies from general healthcare contexts were included only when they addressed appraisal processes, rater behavior, or measurement properties relevant to nursing management; studies focused exclusively on patient outcomes were excluded.

### 2.4. Study Selection and Data Extraction

Screening was conducted by the first author (ET). A second reviewer (NF) independently screened a 20% random sample of titles and abstracts to assess consistency. The second reviewer agreed with the first author’s screening decisions in 94% of cases (184/196 records); the remaining 12 records (6.1%) were disagreements. Agreement was calculated as simple percentage agreement (number of agreed decisions divided by total decisions in the sample). This statistic does not account for chance agreement, and a chance‐corrected statistic (Cohen’s kappa) was not calculated; this is acknowledged as a limitation. Disagreements primarily concerned studies addressing appraisal in healthcare administration contexts without direct nursing samples; these were resolved through discussion, and a third reviewer (MA) was consulted when consensus could not be reached. Full‐text assessment was conducted by the first author, with uncertain cases discussed with the co‐authors. Data extraction was performed by the first author and independently verified by the second reviewer for a 25% random sample of included studies. The 25% sample was selected using a computer‐generated random number list applied to the list of 51 included sources. Because the extraction form included the evidence category and domain, these classifications were verified together with the other extracted data. Discrepancies were resolved by discussion between the first and second reviewers; when consensus could not be reached, the third reviewer adjudicated. No agreement statistic was calculated for the extraction verification.

A standardised data extraction form was used to record author, year, country, design, sample, setting, evidence category, domain, main finding, relevance to the model and limitations. Supporting Table S1 (Supporting File 3) presents a summary of the 51 included studies, including author, publication year, country, study design, sample characteristics, setting, principal findings, methodological limitations and relevance to the proposed framework. Supporting Table S1 lists 53 reference entries: the 51 included sources and the two SANRA methodological references [40, 41], which are listed for completeness and are not counted as evidence. Entries [11, 14, 15, 22, 28–32, 36, 42–44] comprise seminal and methodological works identified through reference‐list checking and citation tracking, together with the Standards [22], and are cited in the text where they inform the framework.

Reporting quality was guided by the Scale for the Quality Assessment of Narrative Review Articles (SANRA) [40, 41]. SANRA is a six‐item tool intended to support authors in planning and reporting narrative reviews and to help reviewers and editors judge them. It was applied here by the authors as a self‐assessment (Supporting File 1) and does not constitute an independent or external quality rating; the self‐assessed scores should be read as an indication of reporting transparency rather than of the validity of the synthesis.

### 2.5. Evidence Classification and Critical Appraisal Strategy

To prevent the integrated model from appearing to overclaim, evidence was classified along two dimensions. The first dimension concerned relevance: direct evidence in acute‐care nursing (studies conducted in acute care or hospital nursing settings), evidence from nursing outside acute care (nursing home, community, or other nonacute nursing settings) and evidence from general performance‐assessment research (non‐nursing organizational contexts or general assessment methodologies). The second dimension concerned evidence characteristics: sample size, study design, methodological quality, directness to nursing and relevance to the proposed mechanism. This classification is an analytic framework created by the authors rather than a validated evidence hierarchy. Directness, sample size, design, methodological quality and nursing relevance were considered separately. Where a proposition is supported primarily by evidence from lower tiers or from non‐nursing contexts, this is explicitly noted. The classification was applied during data extraction and verified during synthesis; it informed, but did not mechanically determine, the weight given to each source. Table 1 summarises, for each pathway in the proposed model, the evidence basis, the main sources and what is and is not established; Table 2 summarises the composition of the 51 included sources by evidence relevance, study design and domain.

**TABLE 1:** Evidence basis for each proposed relationship.

Relationship	Evidence basis	Main sources	What is established	What is not established
Rater behavior ⟶ Score information	Direct large‐scale empirical	Dexter et al. [10]; Dexter et al. [9]	Uniform rating produces measurable information loss; raw ranks misclassify 88% of ratees (nurse anesthetists) versus leniency‐adjusted ranks within the nurse‐anesthetist evaluation context	Prevalence in general acute‐care nursing; effects of specific rater training
Rater behavior ⟶ Score variance	Indirect empirical	Paramesh et al. [8]	One‐third of rating variance attributable to rater‐specific factors	Prevalence in nursing contexts
Agreement ≠ Accuracy	Direct nursing empirical (nonacute)	Sheridan et al. [2]	High agreement can coexist with leniency and halo	Contemporary replication in acute care
Reliability and differentiation	Methodological evidence	Sijtsma & Pfadt [3]; O’Brien et al. [6]	Reliability coefficients do not by themselves establish that scores provide adequate differentiation	Direct demonstration in nursing appraisal
Appraisal system design ⟶ Rater motivation and cognition	Theoretical; indirect	Levy & Williams [18]; Speer et al. [45]; Tenbrink & Speer [46]	Rater motives and perceived accountability are measurable constructs	That specific design features (criteria, anchors, frequency, number of raters) change rater motivation
Rater motivation and cognition ⟶ Rating behavior	Theoretical; indirect (scale validation)	Speer et al. [45]; Polat et al. [47]; Longenecker et al. [20]; Spence & Keeping [21]	Raters report distinct motives; conscious rating distortion is documented	That specific motivation profiles predict specific rating behaviors in nursing
Measurement properties ⟶ Fairness perceptions	Theoretical; indirect	Erdogan [19]; AERA et al. [22]; Kuntarti & Rahmayanto [48]	Fairness perceptions are associated with work outcomes in nursing; theory proposes a link to score interpretation	That score properties cause fairness perceptions (no direct evidence in nursing)
Fairness perceptions ⟶ Future rater motivation and behavior	Theoretical	Erdogan [19]; Colquitt et al. [24]	Fairness perceptions are associated with appraisee attitudes and outcomes	Any reciprocal effect on subsequent rater motivation and behavior in nursing

**TABLE 2:** Architecture of the 51 included sources.

Category	Number of studies
Direct acute‐care nursing	15
Nursing outside acute care	5
General performance assessment	31
Empirical studies	31
Systematic reviews and meta‐analyses	5
Conceptual/theoretical/methodological papers	15
Addressing rater behavior	22
Addressing fairness perceptions	17
Addressing psychometric properties	12

*Note:* Sources were assigned to one primary domain for classification purposes; therefore, the domain counts (22 + 17 + 12) sum to the 51 included sources. Relevance and design counts were derived from Supporting Table S1 and exclude the two SANRA methodological references [40, 41]. Nursing outside acute care includes nursing home, nursing education and nursing‐focused reviews not specific to acute care; general performance assessment includes non‐nursing organizational, educational, psychometric and general healthcare‐worker evidence. Empirical studies include primary‐data studies, including validation and scale development studies; systematic reviews include meta‐analyses; conceptual/theoretical papers include narrative reviews, standards, guidelines and methodological consensus papers.

### 2.6. Conceptual Synthesis and Framework Development

Narrative synthesis was organised by the three core domains, followed by integrative cross‐domain analysis. The synthesis process involved extraction of findings from included studies, classification of findings into fairness, rater behavior and measurement domains, identification of mechanisms connecting domains, comparison of convergent and divergent evidence, identification of recurring causal or reciprocal patterns, development of the proposed framework and distinction between evidence‐supported relationships and theoretical propositions. Evidence from organizational psychology and performance‐assessment research was included when it directly informed mechanisms of rater cognition, rating behavior, measurement error, or appraisal justice, but such evidence was distinguished from nursing‐specific empirical findings during synthesis. Methodological limitations were critically considered during narrative synthesis, with attention to study design, sampling, measurement properties, rater effects and applicability to acute care nursing.

The synthesis was guided by the three research questions. First, studies addressing methods and approaches for evaluating rating quality were identified and categorised by measurement tradition (reliability/agreement, measurement models, rater effect frameworks). Second, studies providing direct empirical evidence on rater behavior and score information were extracted and critically appraised for design, sample and relevance to nursing. Third, studies addressing fairness perceptions and their relationship to score properties were synthesised, with explicit attention to the distinction between empirically established associations and theoretically proposed mechanisms. Cross‐domain integration involved identifying convergent mechanisms and constructing the proposed framework with explicit designation of evidence status for each relationship. Convergent evidence was defined as independent studies pointing to the same mechanism or association; divergent evidence was defined as findings that qualified, contradicted, or limited the generality of a proposed relationship. Divergent findings are reported explicitly in the Results and reflected in the boundary conditions in Table 3.

**TABLE 3:** The three propositions: evidence status and testing requirements.

Proposition	Evidence status	Main evidence	Boundary conditions	Required test
1. Rater behavior alters score information	Empirically supported (direct)	Dexter et al. [9, 10]; Paramesh et al. [8]; Sheridan et al. [2]	Nurse‐anesthetist context; uniform rating; information‐theoretic measures	Replication in general acute‐care nursing; test effects of rater training
2. Consequences depend on differentiation requirements	Theoretical/methodological	G‐theory [6, 9]; absolute vs relative decisions	Purpose of appraisal; competency verification vs ranking	Comparative study of appraisal systems with different purposes
3. Score properties ⟶ perceived fairness depends on interpretation of credibility/legitimacy	Theoretical; requires direct testing	Erdogan [19]; Colquitt [23]; AERA et al. [22]	Adequacy of explanations; rater credibility; developmental stage	Direct empirical test in acute‐care nursing linking score properties to fairness perceptions

## 3. Results

The Results are organised according to the three research questions. Research Question 1 addresses the methods used to evaluate rating quality. Research Question 2 addresses what the most direct evidence establishes about rater behavior and score information. Research Question 3 addresses fairness and its relationship to score properties; the integrative framework that completes this question is presented in the Discussion. Evidence is reported by evidence category (Table 1 and Supporting Table S1), and each critical appraisal statement identifies what a body of evidence does and does not establish.

### 3.1. Research Question 1: What Methods and Approaches are Used to Evaluate Rating Quality?

The reviewed literature employs three principal approaches to evaluating rating quality: reliability and agreement indices, measurement models and rater effect frameworks. These approaches are complementary rather than interchangeable, and each can establish some properties while leaving others unaddressed.

Reliability and agreement approaches include internal consistency coefficients (e.g., Cronbach’s alpha), inter‐rater reliability indices and generalizability coefficients. These approaches quantify the consistency of ratings. They can establish whether ratings are internally consistent or in agreement across raters or occasions. They cannot, by themselves, establish whether ratings are accurate, whether they differentiate performance adequately, or whether they are valid for a particular use. Sijtsma and Pfadt [3] demonstrated that alpha alone is insufficient evidence of measurement quality. O’Brien et al. [6] applied Generalizability Theory to nursing performance assessment, demonstrating that multiple sources of measurement error contribute to ratings and that agreement alone does not adequately capture reliability. Bjork et al. [7] found that measurement error was mainly caused by raters rather than items, recommending multiple raters to improve reliability.

Measurement models, including many‐facet Rasch measurement and mixture Rasch facets models, provide tools for distinguishing genuine performance differences from rater effects. They can establish the structure and magnitude of variance sources, including rater effects, and can test whether criterion order or rater severity influences ratings. Kim [4] demonstrated that criterion order influences halo effects in rating, and Jin and Chiu [5] developed a mixture Rasch facets model capable of distinguishing genuine similarity across dimensions from illusory halo produced by rater bias.

Rater effect frameworks focus on documenting and quantifying the contribution of raters to observed score variation. They can establish the share of rating variance attributable to raters and, in information‐theoretic applications, the amount of information lost through uniform rating. Paramesh et al. [8] found that approximately one‐third of rating variation was attributable to idiosyncratic rater factors. The nurse‐anesthetist studies by Dexter et al. [9, 10] employed information‐theoretic measures to quantify information loss directly.

These three approaches address different aspects of rating quality. Reliability coefficients address consistency; measurement models address the structure of rater effects; rater effect frameworks address the magnitude and consequences of rater contributions to scores. None of the three, taken alone, establishes accuracy or validity. Table 4 summarises the three approaches, what each can show, and the limits of the available evidence in nursing.

**TABLE 4:** Approaches to evaluating rating quality: what each can and cannot show.

Approach	What it evaluates	Representative sources	What it can show	What it cannot show/limits of evidence in nursing
Reliability and agreement indices	Consistency of scores across items, raters, or occasions (e.g., Cronbach’s alpha, inter‐rater indices)	Sijtsma & Pfadt [3]; Sheridan et al. [2]; Polat et al. [47]	Whether ratings are internally consistent or in agreement	Agreement can coexist with leniency and halo; coefficients do not show accuracy or differentiation
Measurement models (G‐theory; many‐facet and mixture Rasch)	Structure and magnitude of variance sources, including rater effects	Kim [4]; Jin & Chiu [5]; O’Brien et al. [6]; Bjork et al. [7]	Whether score variance reflects raters rather than ratees; effects of criterion order; design implications	Largely from nursing education or non‐nursing assessment settings; not applied to acute‐care appraisal scores in practice
Rater‐effect frameworks	Magnitude and consequences of rater contributions to observed scores	Paramesh et al. [8]; Dexter et al. [9, 10]; Odetunde [11]	Share of rating variance attributable to raters; information loss and rank misclassification from uniform rating	Strongest evidence comes from nurse‐anesthetist evaluations at one institution and from general organizational samples; prevalence in general acute‐care nursing is unknown

*Note:* Sources are listed by primary relevance to each approach; study‐level details and methodological limitations are given in Supporting Table S1 (Supporting File 3).

### 3.2. Research Question 2: What is Actually Established About Rater Behavior?

#### 3.2.1. The Strongest Evidence: Large‐Scale Demonstration of Information Loss

The most direct empirical evidence that raters alter the information contained in performance scores comes from a series of studies on nurse anesthetist evaluations. Dexter et al. [10] analyzed 40,027 evaluations of nurse anesthetists over 5.8 years. They found that most loss of information originated from raters who provided all ratees with the largest possible score for all items and from raters who never provided ratings with the maximum score. Excluding 33 raters who provided all ratings as 1s or 0s, the remaining 41 raters had median binomial entropies that were 76% of the normalised maximum entropy. This finding demonstrates that uniform rating behavior produces measurable information loss within the nurse‐anesthetist evaluation system studied.

The practical consequences of this information loss were demonstrated by Dexter et al. [9], who found that raw, unadjusted ranks falsely misclassified 88% of nurse anesthetists. This finding should be interpreted within the specific statistical methods and comparator used in the original study. The 88% misclassification rate reflects the discrepancy between unadjusted ranks and leniency‐adjusted ranks within the specific nurse‐anesthetist evaluation system examined. It does not represent an independently verified error rate in actual clinical competence or personnel decisions. The finding demonstrates that rater leniency can substantially alter relative rankings; it does not establish that 88% of nurses are misclassified in actual clinical competence or that 88% of nursing appraisal systems produce incorrect decisions.

Critical appraisal: The nurse‐anesthetist evidence is powerful because of the very large number of evaluations, the longitudinal design and the use of information‐theoretic measures that quantify information loss directly. However, its transferability to general acute‐care nursing performance appraisal is limited by the specific occupational and supervisory context. The finding that uniform rating produces information loss is likely generalizable as a mechanism, but the prevalence of uniform rating behavior across nursing specialties remains unknown. This evidence establishes the mechanism; it does not establish its prevalence in all nursing contexts.

#### 3.2.2. Supporting Evidence: Idiosyncratic Rater Effects and Halo

Paramesh et al. [8] found that approximately one‐third of rating variation was attributable to idiosyncratic rater factors, including similarity in personality and workplace identities. When a substantial proportion of rating variance reflects the rater rather than the ratee, observed scores may represent rater characteristics as well as ratee performance.

Critical appraisal: This finding is from general performance assessment rather than acute‐care nursing. It supports the mechanism that rater‐specific variance is substantive, but it does not establish the prevalence of idiosyncratic rater effects in nursing.

Sheridan et al. [2] demonstrated in a study of ten nursing homes that behaviorally anchored rating scale (BARS) evaluations showed significant convergent reliability between independent raters but suffered from leniency clustering and halo effects. This finding illustrates that agreement and internal consistency can coexist with systematic rater distortion. High agreement may reflect shared bias rather than accurate measurement.

Critical appraisal: The nursing‐home evidence demonstrates that leniency and halo can occur in nursing assessment, but its age and setting limit its ability to establish how contemporary acute‐care appraisal systems operate. Contemporary replication in acute care is needed.

Kim [4] demonstrated through many‐facet Rasch measurement that criterion order influences halo and that halo produces overly similar ratings across dimensions, compromising diagnostic information. Jin and Chiu [5] developed a mixture Rasch facets model capable of distinguishing genuine similarity across dimensions from illusory halo produced by rater bias.

Critical appraisal: These methodological studies establish that halo can be distinguished from genuine similarity and that criterion order is a design feature that can influence rating behavior. They provide tools for future nursing research rather than direct evidence about nursing appraisal.

#### 3.2.3. What This Evidence Establishes

The evidence reviewed above indicates that rater behavior can function as a substantive component of score production rather than merely as unwanted statistical variance. Systematic rating behavior can change the information contained in scores, which can have implications for decisions made from those scores. The most direct empirical evidence comes from large‐scale nurse‐anesthetist evaluations; supporting evidence from general performance assessment and older nursing‐home studies establishes the mechanism but not its prevalence in contemporary acute‐care nursing.

The evidence does not establish that rater behavior leads to fairness perceptions. It does not establish that the information loss demonstrated in nurse‐anesthetist evaluations occurs with similar prevalence in general medical‐surgical, critical care, or emergency nursing. It does not establish that rater training or system design interventions can reduce information loss in nursing contexts.

#### 3.2.4. Rater Motivation as an Explanatory Mechanism

Rater motivation has been operationalised by Speer et al. [45], who developed and validated the Performance Appraisal Motivation Scale (PAMS), identifying five rater motives: accuracy, conflict avoidance, coaching for improvement, encouragement and system gaming. Tenbrink and Speer [46] subsequently developed the Rater Accountability Scale, demonstrating that raters’ perceived accountability influences their appraisal behavior. Polat et al. [47] confirmed that rater motivation is measurable in nursing management samples, validating the Turkish version of the Performance Appraisal Motivation Scale with 563 nurse managers (overall Cronbach’s alpha = 0.932).

Critical appraisal: These scale‐validation studies establish that rater motivation is a measurable construct in nursing management. They do not establish that specific motivation profiles predict specific rating behaviors, nor that rating behaviors predict score information in nursing contexts. Scale validation establishes measurability, not causal effects.

### 3.3. Research Question 2 (Continued): What is Actually Established About Score Information?

#### 3.3.1. Restriction of Range and Differentiation

Central tendency and restriction of range occur when raters do not use the full scale, so variance is restricted and the instrument loses its capacity to differentiate performance. The nurse‐anesthetist evidence demonstrates that this is a measurable phenomenon with practical consequences [9, 10]. Halo effect reduces discriminant reliability by producing overly similar ratings across dimensions [4, 5]. Leniency bias produces scores that cluster in upper scale ranges, reducing differentiation among ratees [2]. When ratings are uniformly high, differentiation is absent.

Critical appraisal: The relationship between restricted variance and criterion‐related validity is not uniform across all psychometric properties [3]. Restricted variance necessarily affects differentiation, but restricted variance does not automatically mean that an instrument lacks validity. Reduced score variability may result from systematic rating behavior (e.g., leniency, central tendency) or may reflect genuine similarities in employee performance. Similarly, restricted score distributions should not automatically be interpreted as evidence of poor validity without considering the intended measurement purpose and available external criteria. For example, a competency verification system may appropriately produce uniformly high ratings if all assessed nurses genuinely meet the required standards. The evidence establishes that restricted variance is a measurable phenomenon with practical consequences for differentiation; it does not establish a simple linear relationship between variance and validity. It is therefore important to distinguish among reliability, differentiation, information, validity, accuracy and fairness, which are related but nonequivalent concepts.

#### 3.3.2. Reliability and Agreement Can Coexist With Bias

Internal consistency was demonstrated by the Performance Appraisal Motivation Scale Turkish version, which achieved an overall Cronbach’s alpha of 0.932 in a sample of 563 nurse managers [47]. A work habits scale used for daily evaluation of nurse anesthetists demonstrated internal consistency of alpha equal to 0.952 [49]. However, Sijtsma and Pfadt [3] demonstrated why alpha alone is insufficient evidence of measurement quality. Inter‐rater reliability was demonstrated in BARS evaluations, which showed significant convergent reliability between independent raters in nursing home settings, but leniency clustering suggests this may reflect shared bias rather than true agreement [2]. O’Brien et al. [6] demonstrated that rater agreement alone does not adequately capture reliability because multiple sources of measurement error contribute to ratings. Bjork et al. [7] found that error of measurement was mainly caused by raters, not items, and recommended using multiple raters.

Critical appraisal: The coexistence of high agreement and systematic bias is theoretically important. It means that reliability coefficients cannot be interpreted as evidence that ratings are accurate or that they differentiate performance. This finding has direct implications for nursing management: a reliable appraisal instrument may still produce scores that fail to differentiate nurses, or that systematically misrepresent performance. Reliability, agreement and systematic bias are distinct properties; high internal consistency or inter‐rater agreement does not demonstrate accuracy or validity.

#### 3.3.3. Generalizability Theory and Decision Consequences

Generalizability Theory was applied by O’Brien et al. [6], who found that ratings contain multiple sources of measurement error. Evidence from nurse‐anesthetist evaluations demonstrated that even when very high generalizability coefficients (G‐scores) of 0.90 are obtained, ranking individuals into smaller groups such as deciles, or using raw observed ranks, is unreliable [9].

Critical appraisal: The distinction between reliability for absolute decisions and reliability for relative decisions is crucial. High G‐scores may support absolute decisions (e.g., competent/not competent) while being insufficient for relative decisions (e.g., ranking for promotion). Nursing management must therefore specify the intended use of appraisal scores before selecting reliability thresholds.

#### 3.3.4. Content and Construct Validity in Nursing Instruments

Content validity is frequently established through expert panels, and COSMIN methodology provides structured criteria for evaluating content validity [12–15, 43]. Ta’an et al. [34] found that the Arabic version of the Six‐Dimension Scale demonstrated acceptable model fit, but exploratory factor analysis identified a three‐factor solution despite the original six‐dimensional conceptualization. Sugimura et al. [35] found that confirmatory factor analysis supported a three‐factor model for the Japanese version of the Nursing Performance Instrument, but internal consistency was insufficient.

Critical appraisal: The contrast between Ta’an et al. [34] and Sugimura et al. [35] indicates that different versions of nursing‐performance instruments may show acceptable structural validity while producing different dimensional structures and different reliability profiles across contexts. This finding suggests that structural validity is context‐dependent and that cross‐cultural adaptation may alter the construct being measured.

#### 3.3.5. Criterion Validity: What Should Appraisal Scores Predict?

Krijgsheld et al. [50] demonstrated that healthcare job performance is multidimensional, encompassing task, contextual, adaptive and counterproductive performance. Kuntarti and Rahmayanto [48] found no significant association between nurses’ formal performance appraisal scores and caring behavior in a sample of 228 hospital nurses.

Critical appraisal: The finding of no association between appraisal scores and caring behavior raises fundamental questions about construct representation. If appraisal scores do not reflect clinically valued behaviors, then their validity for nursing management decisions is questionable. This finding does not establish that appraisal scores are invalid for all purposes, but it does establish that criterion validity cannot be assumed and must be demonstrated for specific intended uses.

Sucipto et al. [37] systematically reviewed 13 studies of nurse performance appraisal instruments published between 2020 and 2025. They identified persistent concerns including subjectivity, inconsistent benchmarks, limited validation, fairness and communication.

Critical appraisal: The systematic review by Sucipto et al. [37] establishes that the field has not resolved fundamental measurement challenges. However, the review did not examine how rater behavior affects the instruments in practice, nor did it link measurement properties to fairness perceptions. This is the gap that the present review addresses.

### 3.4. Research Question 3: What is Actually Established About Fairness?

#### 3.4.1. Nursing Evidence on Fairness Perceptions

Nurses view performance appraisal as an essential tool for professional development, not merely an administrative formality. Rabenu and Shertzer [38] conducted a qualitative study of 34 nurses in Israel and identified four themes: feedback session, appraisee, appraiser and appraisal process. Jerónimo and Araújo [51] identified structural and process problems in nursing performance appraisal. Jaber et al. [39] examined 356 nurses in a tertiary hospital and found significant differences in perceptions of appraisal efficacy across nursing roles.

The evidence consistently demonstrates positive relationships between perceived appraisal fairness and desirable organizational outcomes. With respect to work engagement, Hamdeen et al. [25] found a positive association between perceived appraisal fairness and work engagement among 250 staff nurses. Brefo‐Manuh and Anlesinya [26] demonstrated in 302 healthcare workers that interpersonal and procedural appraisal justice were associated with job satisfaction, while distributive and procedural justice predicted organizational commitment. Sarhan et al. [52] found a significant positive relationship between appraisal fairness and nurses’ work motivation among 280 staff nurses in Egypt. Rubel and Kee [53] found that perceived appraisal fairness and promotion opportunity were associated with organizational commitment and lower turnover intention among 150 full‐time nurses in private hospitals. Joseph and Varghese [27] reviewed 11 cross‐sectional studies and concluded that structured annual appraisals incorporating clear expectations, constructive feedback, fairness and transparency and professional development were associated with improved job performance and satisfaction.

#### 3.4.2. Critical Appraisal: What Fairness Studies Do Not Establish

The nursing fairness studies reviewed above provide substantial evidence that perceived appraisal fairness is associated with important work outcomes, thereby establishing the organizational relevance of fairness perceptions. However, they do not establish that the psychometric properties of ratings cause those fairness perceptions. These studies are predominantly cross‐sectional and correlational and therefore cannot establish causal direction; they measure employees’ fairness perceptions without simultaneously modeling score distributions or rater behavior; they do not examine whether nurses who receive more differentiated ratings perceive greater fairness; they do not link specific rater behaviors to specific fairness judgments encompassing procedural, interpersonal, informational and distributive dimensions; and they do not establish that improving measurement properties would improve fairness perceptions.

The relationship between score properties and perceived fairness remains theoretically plausible but empirically untested in nursing. Erdogan’s [19] framework identified appraisal ratings as antecedents of justice perceptions, but direct empirical evidence linking measurement properties to fairness perceptions in acute care nursing is limited.

#### 3.4.3. The Tension Between Measurement Quality and Fairness

It is possible for an appraisal system to be experienced as fair while producing poor measurement information, and conversely, for a psychometrically differentiated system to be experienced as unfair. Leniency may increase interpersonal satisfaction while reducing informational value: a nurse manager who gives uniformly high ratings may be perceived as supportive and interpersonally fair, while simultaneously depriving nurses of meaningful developmental feedback. Uniform ratings might appear supportive to some employees but undermine perceptions of differentiation or credibility. Strict ratings could increase differentiation while being perceived as unfair if procedures or explanations are inadequate. These tensions suggest that measurement quality and fairness are related but nonequivalent dimensions of appraisal system quality. Improving one does not necessarily improve the other.

#### 3.4.4. What Justice Theory Contributes

Colquitt [23] established four distinct dimensions of justice that remain the standard taxonomy: distributive justice, procedural justice, interpersonal justice and informational justice. Colquitt et al.’s [24] meta‐analysis of 183 justice studies demonstrated that these four dimensions are related but provide incremental explanatory value for organizational outcomes.

#### 3.4.5. What Measurement Theory Contributes

Measurement theory directs attention to the informational properties of ratings: whether scores differentiate performance, whether they contain sufficient variance to support decisions, and whether they represent ratee behavior or rater characteristics. A rating system that produces uniformly high scores may be perceived as procedurally fair in its administration yet fail to provide the differentiation necessary for distributive justice in outcomes.

Critical synthesis: Justice research examines fairness perceptions and their consequences but rarely addresses rater cognition or measurement precision. Measurement research documents rating errors and their correlates but seldom connects to how nurses perceive the fairness of the resulting evaluations. This separation reflects an implicit assumption that fairness, rater behavior and measurement quality are analytically separable problems that can be addressed independently. The evidence reviewed in this article suggests that this assumption may be untenable, but the relationships require direct empirical testing before they can be treated as established.

#### 3.4.6. The Missing Link: Score Information and Perceived Fairness

Several theoretical mechanisms may plausibly connect score properties with fairness perceptions, and these are presented here as hypotheses rather than as mechanisms supported by the nursing evidence. First, with respect to differentiation and distributive justice, if ratings do not differentiate performance, nurses who observe underperforming colleagues receiving identical ratings may perceive distributive injustice because the outcome does not reflect the input. Second, with respect to information content and informational justice, if ratings do not provide meaningful information about performance, nurses may perceive the process as lacking informational justice. Third, with respect to credibility and procedural justice, if ratings are uniformly high or systematically distorted, nurses may question the credibility of the appraisal process. Fourth, with respect to feedback utility and interpersonal justice, if ratings do not provide useful developmental feedback, nurses may perceive the appraisal interaction as disrespectful.

Direct empirical evidence linking measurement properties to fairness perceptions in acute care nursing is limited. The nursing fairness studies reviewed above measure perceptions without modeling score distributions or rater behavior. Indirect evidence comes from Kuntarti and Rahmayanto [48], who raise the question of whether appraisal scores correspond to the behaviors nurses and patients value. However, this study did not directly measure fairness perceptions or test the relationship between score properties and fairness.

The relationship between score information and fairness perceptions matters for nursing management because it determines whether interventions should focus on rater behavior, instrument design, fairness perceptions, or all three. The reviewed literature supports examining whether the relationship is reciprocal and contingent; direct testing in nursing contexts is required.

## 4. Discussion

### 4.1. Rater Behavior as an Enactment Condition

The evidence reviewed above supports a reframing of the rater in nursing performance appraisal. Rater behavior is a constitutive component of the process through which performance‐assessment scores are produced. Rater motivation is hypothesised to shape rating behavior; the available scale‐validation evidence [45–47] shows that rater motives are measurable, not that they cause specific rating patterns. Rater behavior is proposed to affect psychometric properties, with direct evidence currently limited to score information and ranking [9, 10]. Leniency, halo and central tendency alter the distribution of observed scores. Evidence from nurse‐anesthetist evaluations shows that raters who provide uniform ratings contribute disproportionately to information loss [10].

This reframing has implications for how validity is conceptualised. According to contemporary psychometric frameworks [17, 22], validity is not a property of an assessment instrument but of score interpretations for specific uses. Evidence for validity cannot be interpreted independently of the conditions under which scores are generated, interpreted and used. If rater behavior systematically alters the information contained in scores, then validity cannot be fully understood at the instrument level alone. It must also consider how the instrument is enacted by raters within an organizational context. The distinction between instrument validity and validity of the assessment process in use is central to this review.

The concept of ‘enactment condition’ advances existing theories in three ways. Relative to rater‐effect theories, it shifts attention from the magnitude of rater variance to the consequences of rater behavior for the informational properties of scores. Relative to measurement theories, it treats rater behavior as part of the conditions under which validity evidence is generated, rather than as an extraneous source of error. Relative to justice theories, it identifies a testable pathway from score properties to fairness perceptions that has not been directly examined in nursing. The concept is not a new measurement property, and it does not imply that raters are the sole determinant of validity; it is a conceptual synthesis that organises existing evidence and identifies unresolved relationships.

### 4.2. Three Propositions

#### 4.2.1. Proposition 1: Rater Behavior Constitutes an Enactment Condition of Performance Measurement, Such That Systematic Rating Behavior Can Alter the Informational Properties of Performance Scores

This is the strongest proposition, supported by large‐scale empirical evidence from nurse‐anesthetist evaluations [9, 10] and supporting evidence from general performance assessment [8] and nursing‐home studies [2]. The evidence establishes that uniform rating behavior produces measurable information loss and that raw ranks can misclassify ratees. The mechanism is established; the prevalence in general acute‐care nursing requires direct investigation.

#### 4.2.2. Proposition 2: The Consequences of Rater Behavior are Expected to Depend on the Degree to Which the Assessment System Requires Differentiation Among Ratees and Performance Dimensions

This proposition is theoretically and methodologically derived; it is not directly tested in nursing. Not all appraisal systems require differentiation. A system used solely for formative feedback may function adequately with restricted variance. A system used for promotion, retention, or competency verification requires differentiation. A critical distinction must be made between relative performance comparisons and criterion‐referenced competency assessments. Promotion decisions based on comparative performance may require differentiation among employees, whereas competency verification may primarily require determining whether predefined professional standards have been achieved. In the latter context, similar or uniformly high ratings may be appropriate if the assessed employees genuinely meet the required standards. Greater score differentiation is not necessarily an indicator of improved measurement quality. The adequacy of score information should instead be evaluated in relation to the intended purpose of the assessment and the decisions supported by the scores. This proposition is supported by the distinction between absolute and relative decisions in Generalizability Theory [6, 9] and by the finding that high reliability for absolute decisions does not guarantee reliability for relative decisions. It is not, however, empirical evidence that nursing managers respond differently to rater behavior depending on the purpose of the appraisal system.

#### 4.2.3. Proposition 3: The Relationship Between Score Properties and Perceived Appraisal Fairness Depends on How Nurses Interpret the Credibility and Legitimacy of Score Differentiation

This proposition is explicitly presented as requiring empirical testing. The theoretical basis is strong [19, 23], but direct empirical evidence in acute care nursing is lacking. Contemporary fairness frameworks [22] conceptualize fairness as encompassing equitable treatment, absence of construct‐irrelevant variance and appropriate score interpretation. These frameworks provide a basis for examining how score properties may relate to fairness perceptions, but direct empirical evidence in nursing contexts is required. Informational adequacy may function as a moderator and is discussed in the text rather than embedded in the proposition. The relationship is likely contingent on multiple factors, including the adequacy of explanations, the perceived credibility of the rater and the developmental stage of the nurse. Table 3 summarizes the evidence status, boundary conditions and required test for each proposition.

Figure 2 presents the integrated conceptual model that links the three propositions. The model does not claim that the reciprocal relationship between fairness perceptions and subsequent rater behavior is empirically established in nursing, that measurement quality and fairness are equivalent or that improving one necessarily improves the other, that the model has been tested as a causal model, or that the propositions are equally well supported by evidence. Rather, the model distinguishes empirically supported relationships (Proposition 1) from relationships requiring direct testing (Propositions 2 and 3). This distinction prevents the model from overclaiming and identifies specific empirical designs required to test unresolved relationships in nursing contexts.

**FIGURE 2:**
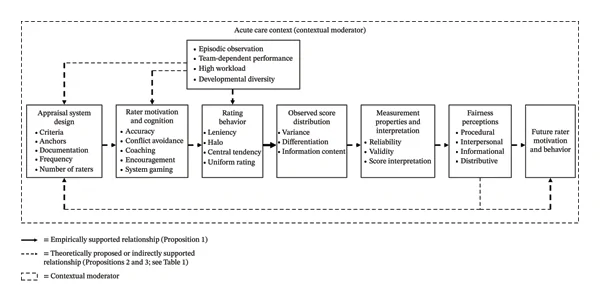
Rater‐Enacted Assessment System Model. The figure was created originally by the authors; no copyright permission is required. The model links appraisal system design to rater motivation and cognition, rating behavior, observed score distributions, measurement properties and fairness perceptions, which may influence future rater motivation and behavior. Appraisal design includes criteria, anchors, documentation, frequency and number of raters; rater behavior includes leniency, halo, central tendency and uniform rating. Perceived appraisal justice comprises distributive, procedural, interpersonal and informational dimensions. The acute care context may moderate these relationships through episodic observation, team‐dependent performance, high workload and developmental diversity. Only one pathway—rating behavior to observed score distribution—is drawn as a solid arrow because it is supported by empirical evidence (Proposition 1). All other pathways, including appraisal system design to rater motivation and cognition, rater motivation and cognition to rating behavior, observed score distribution to measurement properties, measurement properties to fairness perceptions, fairness perceptions to future rater motivation and behavior and the contextual moderation pathways, are drawn as dashed arrows because they are theoretically proposed or only indirectly supported and require direct testing (Propositions 2 and 3). The dashed feedback arrow from Fairness Perceptions back to Appraisal System Design indicates a theoretically proposed reciprocal relationship, wherein perceived fairness may influence subsequent modifications to the appraisal system. The level and context of evidence supporting each pathway are identified in Tables 1 and 3. The surrounding box represents the contextual moderator.

## 5. Implications for Nursing Management

### 5.1. For Nurse Managers as Raters

Lenient or uniform rating can reduce the differentiation and developmental information available from appraisal scores. The extent to which this translates into poorer developmental feedback or lower appraisal credibility requires direct empirical evaluation. Nurse managers should be aware that their rating behavior is not merely a private choice; it is an enactment of the measurement system that affects the information available for personnel decisions. Rater training, calibration mechanisms and multiple raters may be considered to ensure that psychometric refinement translates into more informative appraisal practice, but their effects on score information and fairness in nursing appraisal require direct evaluation.

These recommendations should be distinguished by their evidence status. The finding that uniform rating produces information loss is empirically established [9, 10]. The recommendation to use multiple raters is supported by Generalizability Theory evidence [6, 7] demonstrating that rater variance contributes substantially to measurement error. However, the effectiveness of specific rater training programs in nursing contexts requires direct evaluation; general organizational evidence on the frequency and effects of rater training is available [42, 44], but its transferability to nursing appraisal has not been established. Calibration mechanisms and structured documentation are proposed interventions that have not been empirically tested in nursing appraisal contexts.

### 5.2. For Appraisal System Designers

Validation studies should include indices of rater behavior, such as variance across raters, as part of psychometric reporting, rather than treating rater effects as extraneous. Researchers should distinguish between instrument validity and validity of the assessment process in use. System design features, including number of raters, documentation requirements, feedback frequency and criteria specificity, can either attenuate or amplify rater effects. Structured documentation and behavioral observation could be evaluated as strategies for reducing reliance on retrospective memory. Educative feedback to raters who consistently provide uniform ratings could be evaluated for its effects on score differentiation.

### 5.3. For Organizations

Performance appraisal systems in acute care may be designed with rater training, calibration mechanisms and multiple raters where feasible. Evidence from nurse‐anesthetist evaluations indicates that accounting for rater leniency can improve the reliability of relative ranking within that specific evaluation context [9]. Organizations should recognize that measurement quality and fairness are related but nonequivalent. The recommendations above are evidence‐informed considerations, not proven intervention effects. Greater score differentiation is not always desirable; its value depends on the intended purpose of the appraisal and the decisions being made.

The practical feasibility of these strategies must be considered in healthcare organizations. Workload constraints, limited observation opportunities, and resource limitations may influence the implementation of multiple‐rater systems, structured documentation and calibration mechanisms. Nurse managers in acute care settings often supervise large numbers of staff with limited opportunity for direct observation. These contextual constraints may limit the feasibility of resource‐intensive strategies and should inform the design of appraisal systems that balance measurement quality with practical utility.

### 5.4. Strengths and Limitations

#### 5.4.1. Strengths

This review addresses an under‐researched integration of three bodies of literature that have developed largely in isolation. The use of a two‐dimensional evidence classification provides a transparent framework for distinguishing what is empirically established from what remains theoretically proposed. The explicit critical appraisal of key studies, including what they establish and what they do not establish, prevents the review from overclaiming. The review includes international evidence from Australia, Bangladesh, Canada, Chile, China, Egypt, Germany, Ghana, India, Indonesia, Israel, Japan, Jordan, the Netherlands, Nigeria, Norway, Portugal, Saudi Arabia, South Korea, Spain, Turkey and the United States.

#### 5.4.2. Limitations

Narrative review methodology is interpretive and potentially shaped by author perspective; no formal quantitative risk‐of‐bias scoring or meta‐analysis was performed. The limitations of this review include several factors that may affect the confidence that can be placed in the synthesis and in the proposed framework.

First, single‐reviewer screening for the majority of records may have introduced selection bias. Although a second reviewer independently screened a 20% random sample with 94% agreement, the remaining 80% of screening decisions were made by one reviewer. Agreement was reported as simple percentage agreement; a chance‐corrected statistic (Cohen’s kappa) was not calculated. Similarly, data extraction was independently verified for only a 25% random sample of included studies, and formal agreement statistics (e.g., percentage agreement or Cohen’s kappa) were not calculated for this verification step, so the consistency of extraction beyond the verified sample cannot be quantified. Second, the initial search strategy applied acute‐care and hospital filters in several databases, which may have excluded relevant studies addressing rater behavior and performance appraisal in other organizational settings. Reference‐list checking and citation tracking were used to address this limitation, but these supporting methods may not have captured all relevant studies. A sensitivity search without the acute‐care and hospital filters was not undertaken, so the retrieval of nonacute‐care and non‐nursing evidence depends on these supporting methods. Third, the inclusion of general organizational research, while justified by the transferability criteria, means that some evidence informing the proposed framework derives from non‐nursing contexts. Fourth, no comprehensive study‐level quality assessment using a validated tool was performed; methodological limitations were appraised narratively for key studies. These limitations are relevant to the confidence that can be placed in the proposed synthesis and its applicability across different nursing settings. The literature search may have missed relevant studies in nonindexed journals or non‐English languages. The focus on acute care nursing may limit generalizability to primary care, community, or long‐term care settings. Several propositions in the conceptual model are theoretically derived and require direct empirical testing. The model should therefore be interpreted as a framework for future research rather than as an empirically established causal model. Finally, SANRA was applied as an author self‐assessment (Supporting File 1) and should not be read as an independent quality rating of this review. Taken together, these limitations reduce the confidence that can be placed in the proposed framework and reinforce its status as a testable research agenda rather than a validated causal model.

## 6. Conclusion

Rater behavior, score information and fairness perceptions are interrelated dimensions of appraisal‐system quality in nursing, but the relationships among them are not equally established. The most direct empirical evidence establishes that rater behavior, particularly uniform rating, can alter the informational properties of performance scores, reducing differentiation and producing potentially consequential errors in relative ranking within the specific nurse‐anesthetist evaluation context examined. Evidence that score properties cause fairness perceptions is weaker; nursing fairness research is predominantly cross‐sectional, measures perceptions without modeling score distributions and does not establish that measurement properties cause fairness judgments. The relationship between score information and perceived fairness remains theoretically plausible but empirically untested in nursing.

This review contributes to nursing management by proposing a more limited, testable framework that conceptualizes rater behavior as an enactment condition of performance measurement. Three propositions are advanced: rater behavior systematically alters score information, a proposition with direct empirical support; the consequences depend on whether the system requires differentiation, a primarily theoretical and methodological proposition; and the relationship between score properties and fairness perceptions depends on how nurses interpret the credibility and legitimacy of score differentiation, a proposition requiring direct empirical testing in nursing contexts. These propositions require direct empirical testing in nursing contexts. The framework therefore provides a focused research agenda for examining how assessment instruments function when enacted within real nursing organizations, without claiming that the framework has been empirically validated as a causal model.

## Author Contributions

Ehab Tumeh: conceptualization, methodology, investigation, data curation, formal analysis, writing–original draft, writing–review and editing, project administration. Nehad Fekri: supervision, methodology, independent screening (20% sample), data verification (25% sample), writing–review and editing. Magda Almolla: supervision, methodology, adjudication of screening and data‐extraction discrepancies, writing–review and editing.

## Funding

This research received no specific grant from any funding agency in the public, commercial, or not‐for‐profit sectors. The research was performed as part of the authors’ employment at Cairo University and Nablus University for Vocational and Technical Education.

## Disclosure

All authors approved the final version of the manuscript and agree to be accountable for all aspects of the work.

## Ethics Statement

The authors have nothing to report.

## Consent

The authors have nothing to report.

## Conflicts of Interest

The authors declare no conflicts of interest.

## Supporting Information

Additional supporting information can be found online in the Supporting Information section.

## Supporting information


**Supporting Information 1** Supporting File 1. SANRA Checklist. Author self‐assessment of the reporting quality of this review against the six items of the Scale for the Quality Assessment of Narrative Review Articles (SANRA), with a score (0–2) and a justification for each item.

**Supporting Information 2** Supporting File 2. Complete Database Search Strings. Full search strings used in PubMed/MEDLINE, CINAHL, PsycINFO and Scopus, including the search blocks, controlled vocabulary, truncation and date limits applied in each database.

**Supporting Information 3** Supporting File 3. Supporting Table S1: Evidence Table of Included Studies. Summary of the 51 included sources and the two methodological references on SANRA, presenting author, year, country, design, sample, setting, principal findings, methodological limitations and relevance to the proposed framework.

## Data Availability

Data sharing is not applicable to this article as no new data were created or analyzed in this study. All data discussed are publicly available in the published literature cited in the reference list.
