# Supplementary material for: Rater Behavior as an Enactment Condition in Nursing Performance Appraisal: A Critical Review
Source: J Nurs Manag. 2026 Nov 15;2026:4829440. doi: 10.1155/jonm/4829440 (PMC13770256; doi:10.1155/jonm/4829440)
Supplement: Supplementary file 1 [file JONM-2026-4829440-s001.docx]

**Supplementary File 3. Evidence Table of Included Studies**

**Supplementary Table S1. Summary of the 51 Included Sources and Two Methodological References**

| # | Author (Year) | Country | Design | Sample | Setting | Principal Findings | Methodological Limitations | Relevance to Framework |
| --- | --- | --- | --- | --- | --- | --- | --- | --- |
| 1 | Murphy & Cleveland (1995) [1] | USA | Theoretical | N/A | General organizational | Appraisal as social, cognitive, contextual process | Conceptual; no empirical data | Theoretical foundation |
| 2 | Messick (1989) [2] | USA | Theoretical | N/A | Educational measurement | Validity as integrative evaluative judgment | Conceptual; pre-dates contemporary frameworks | Theoretical foundation |
| 3 | Kane (2013) [3] | USA | Theoretical | N/A | Educational measurement | Validity of score interpretations and uses | Conceptual | Theoretical foundation |
| 4 | Levy & Williams (2004) [4] | USA | Review | N/A | General organizational | Social context of performance appraisal | Narrative review; no systematic search | Theoretical foundation |
| 5 | Erdogan (2002) [5] | Turkey/USA | Theoretical | N/A | General organizational | Rater behavior as cause and consequence of appraisal justice | Conceptual; limited empirical integration | Theoretical foundation |
| 6 | Longenecker et al. (1987) [6] | USA | Qualitative | Managers | General organizational | Conscious rating distortion for political motives | Small sample; dated | Rater motivation |
| 7 | Spence & Keeping (2011) [7] | Australia | Review | N/A | General organizational | Conscious rating distortion framework | Narrative review | Rater motivation |
| 8 | Colquitt (2001) [8] | USA | Construct validation | Multiple samples | General organizational | Four justice dimensions: distributive, procedural, interpersonal, informational | Self-report measures | Justice taxonomy |
| 9 | Colquitt et al. (2001) [9] | USA | Meta-analysis | 183 studies | General organizational | Four justice dimensions provide incremental explanatory value | Meta-analysis of primarily cross-sectional studies | Justice dimensions |
| 10 | Hamdeen et al. (2022) [10] | Egypt | Cross-sectional | 250 staff nurses | Hospital nursing | Positive association between appraisal fairness and work engagement | Cross-sectional; self-report | Fairness → outcomes |
| 11 | Brefo-Manuh & Anlesinya (2023) [11] | Ghana | Cross-sectional | 302 healthcare workers | Healthcare | Interpersonal and procedural justice associated with job satisfaction; distributive and procedural justice predicted organizational commitment | Cross-sectional; self-report | Fairness → outcomes |
| 12 | Joseph & Varghese (2024) [12] | India | Systematic review | 11 cross-sectional studies | Nursing | Structured annual appraisals with clear expectations, feedback, fairness associated with improved performance and satisfaction | All included studies cross-sectional; limited quality assessment | Fairness → outcomes |
| 13 | Paramesh et al. (2020) [13] | India | Cross-sectional | Employee–supervisor dyads | General organizational | One-third of rating variance attributable to rater-specific factors | Cross-sectional; specific industry context | Rater effects |
| 14 | Dexter et al. (2020) [14] | USA | Retrospective analysis | Nurse anesthetist evaluations | Nurse anesthetist evaluation | Raw ranks misclassify 88% of ratees relative to leniency-adjusted ranks; leniency adjustment improves ranking reliability | Specific occupational context; retrospective | Rater behavior → ranking consequences |
| 15 | Dexter et al. (2022) [15] | USA | Retrospective analysis | 40,027 evaluations; 74 raters | Nurse anesthetist evaluation | Uniform rating produces measurable information loss; median binomial entropy 76% of maximum | Specific occupational context; retrospective | Rater behavior → score information |
| 16 | Kim (2020) [16] | South Korea | Many-facet Rasch | Writing assessments | Educational | Criterion order influences halo; halo produces overly similar ratings | Educational context; not nursing | Halo detection methodology |
| 17 | Jin & Chiu (2022) [17] | China | Mixture Rasch facets | Simulation + empirical | Educational | Mixture Rasch facets model distinguishes genuine similarity from illusory halo | Educational context; not nursing | Halo vs. genuine similarity methodology |
| 18 | Schwiran (1978) [18] | USA | Instrument development | Nursing performance | Hospital nursing | Multidimensional nursing performance evaluation | Dated; limited contemporary relevance | Historical instrument development |
| 19 | Ta'an et al. (2024) [19] | Jordan | Validation study | Nursing performance instrument | Hospital nursing | Arabic version demonstrated acceptable fit but three-factor solution vs. original six dimensions | Cross-sectional; single country | Structural validity context-dependent |
| 20 | Sugimura et al. (2023) [20] | Japan | Validation study | Nursing performance instrument | Hospital nursing | Three-factor model supported; internal consistency insufficient | Cross-sectional; single country | Cross-cultural validity |
| 21 | Sucipto et al. (2026) [21] | Indonesia | Systematic review | 13 studies | Nursing | Persistent concerns: subjectivity, inconsistent benchmarks, limited validation | Limited to 2020–2025; English only | Instrument limitations |
| 22 | Rabenu & Shertzer (2025) [22] | Israel | Qualitative | 34 nurses | Hospital nursing | Four themes: feedback session, appraisee, appraiser, process | Small sample; single country | Fairness perceptions |
| 23 | Jaber et al. (2024) [23] | Saudi Arabia | Cross-sectional | 356 nurses | Tertiary hospital | Significant differences in appraisal efficacy perceptions across roles | Cross-sectional; single hospital | Fairness perceptions |
| 24 | Baethge et al. (2019) [24] | Germany | Scale development | N/A | Narrative review methodology | SANRA quality assessment tool | Not empirical | Review quality (methodological reference, not counted among 51) |
| 25 | Fuentes-Barria et al. (2026) [25] | Chile | Methodological | N/A | Narrative review methodology | SANRA guide for narrative reviews | Not empirical | Review quality (methodological reference, not counted among 51) |
| 26 | Sheridan et al. (1983) [26] | USA | Longitudinal | 10 nursing homes | Nursing home | High inter-rater agreement coexists with leniency and halo | Dated; nursing home context | Agreement ≠ accuracy |
| 27 | Speer et al. (2020) [27] | USA | Scale development | Multiple samples | General organizational | PAMS identifies five rater motives | Self-report; general organizational | Rater motivation |
| 28 | Tenbrink & Speer (2023) [28] | USA | Scale development | Multiple samples | General organizational | Rater accountability influences appraisal behavior | Self-report; general organizational | Rater motivation |
| 29 | Polat et al. (2025) [29] | Turkey | Validation study | 563 nurse managers | Hospital nursing | PAMS validated in Turkish nurse managers (α = .932) | Cross-sectional; single country | Rater motivation measurable |
| 30 | Sijtsma & Pfadt (2021) [30] | Netherlands | Methodological | N/A | Psychometric | Alpha alone insufficient evidence of measurement quality | Not empirical | Reliability limitations |
| 31 | Dexter et al. (2017) [31] | USA | Validation study | Nurse anesthetist evaluations | Nurse anesthetist evaluation | Work habits scale internal consistency α = 0.952 | Specific occupational context | Reliability demonstration |
| 32 | O'Brien et al. (2019) [32] | USA | Generalizability Theory | Nursing performance assessment | Nursing education | Multiple sources of measurement error; agreement alone inadequate | Educational context; not clinical | G-theory application |
| 33 | Bjork et al. (2021) [33] | Norway | Generalizability Theory | Practical skill assessment | Nursing education | Error mainly caused by raters, not items; multiple raters recommended | Educational context; not clinical | Rater variance |
| 34 | Terwee et al. (2018) [34] | Netherlands | Delphi study | N/A | Psychometric | COSMIN content validity methodology | Not empirical | Content validity methodology |
| 35 | Gagnier et al. (2021) [35] | Canada | Guideline | N/A | Psychometric | COSMIN reporting guideline | Not empirical | Measurement reporting |
| 36 | Krijgsheld et al. (2022) [36] | Netherlands | Systematic review | Healthcare job performance | Healthcare | Job performance multidimensional: task, contextual, adaptive, counterproductive | Limited to healthcare | Criterion complexity |
| 37 | Kuntarti & Rahmayanto (2026) [37] | Indonesia | Cross-sectional | 228 hospital nurses | Hospital nursing | No significant association between appraisal scores and caring behavior | Cross-sectional; single hospital | Criterion validity questioned |
| 38 | Jerónimo & Araújo (2023) [38] | Portugal | Qualitative | Nurses | Hospital nursing | Structural and process problems in nursing appraisal | Small sample; single country | Fairness perceptions |
| 39 | Sarhan et al. (2023) [39] | Egypt | Cross-sectional | 280 staff nurses | Hospital nursing | Positive relationship between appraisal fairness and work motivation | Cross-sectional; self-report | Fairness → outcomes |
| 40 | Rubel & Kee (2015) [40] | Bangladesh | Cross-sectional | 150 nurses | Private hospitals | Perceived fairness and promotion opportunity associated with commitment and lower turnover intention | Cross-sectional; self-report | Fairness → outcomes |
| 41 | Adamovic (2023) [41] | Australia | Review | N/A | General organizational | Organizational justice research synthesis | Narrative review | Justice review |
| 42 | Cropanzano et al. (2007) [42] | USA | Theoretical | N/A | General organizational | Justice management framework | Conceptual | Justice application |
| 43 | Borba & Spence (2025) [43] | USA | Longitudinal | Raters | General organizational | Rater experience as time-variant predictor of ratings | General organizational | Rater characteristics |
| 44 | Feeney et al. (2024) [44] | Canada | Experimental | Raters | General organizational | Rating accuracy, leniency, and rater perceptions with the Relative Percentile Method (RPM) and behaviorally anchored rating scales (BARS) | Experimental; not nursing | Rating format effects |
| 45 | Greenslade & Jimmieson (2007) [45] | Australia | Scale development | Nurses | Hospital nursing | Task and contextual performance distinction for nurses | Single country | Performance dimensions |
| 46 | Mokkink et al. (2010) [46] | Netherlands | Consensus | N/A | Psychometric | COSMIN taxonomy of measurement properties | Not empirical | Measurement taxonomy |
| 47 | Mokkink et al. (2010) [47] | Netherlands | Checklist development | N/A | Psychometric | COSMIN checklist for methodological quality | Not empirical | Quality assessment |
| 48 | Odetunde (2022) [48] | Nigeria | Scale development | Raters | General organizational | Rater Bias Scale construction and validation | General organizational | Rater bias measurement |
| 49 | Rosales et al. (2021) [49] | Spain | Experimental | Raters | General organizational | Measurement type and timing effects on performance appraisal | Experimental; not nursing | Rating format effects |
| 50 | Speer (2020) [50] | USA | Survey | Organizations | General organizational | Frequency and effects of appraisal training | Survey; not nursing | Rater training effects |
| 51 | Terwee et al. (2007) [51] | Netherlands | Quality criteria | N/A | Psychometric | Quality criteria for measurement properties | Not empirical | Quality criteria |
| 52 | Woehr & Huffcutt (1994) [52] | USA | Meta-analysis | N/A | General organizational | Rater training quantitative review | Dated meta-analysis | Rater training effects |
| 53 | AERA et al. (2014) [53] | USA | Standards | N/A | Educational/psychological measurement | Validity as property of score interpretations for specific uses; fairness as multifaceted concern | Not empirical | Contemporary validity and fairness frameworks |

***Note:*** *Supplementary Table S1 lists 53 entries: the 51 included sources and two methodological references on SANRA ([24], [25]), which are listed for completeness and are not counted as evidence. Reference [53] (Standards for Educational and Psychological Testing) is a theoretical/methodological standard rather than an empirical study. Primary relevance categories were derived from the Setting column: direct acute-care nursing (studies 10, 14, 15, 18, 19, 20, 22, 23, 29, 31, 37, 38, 39, 40, 45; n = 15); nursing outside acute care, including nursing home, nursing education, and nursing-focused reviews (studies 12, 21, 26, 32, 33; n = 5); general performance assessment, including non-nursing organizational, educational, psychometric, and general healthcare-worker evidence (all remaining studies except 24 and 25; n = 31). Sources were assigned to one primary domain for classification purposes; therefore, the domain counts (22 + 17 + 12) sum to the 51 included sources. Abbreviations: AERA, American Educational Research Association; BARS, behaviorally anchored rating scales; COSMIN, COnsensus-based Standards for the selection of health Measurement INstruments; G-theory, Generalizability Theory; PAMS, Performance Appraisal Motivation Scale; RPM, Relative Percentile Method; SANRA, Scale for the Quality Assessment of Narrative Review Articles.*
