# Supplementary material for: Rater Behavior as an Enactment Condition in Nursing Performance Appraisal: A Critical Review
Source: J Nurs Manag. 2026 Nov 15;2026:4829440. doi: 10.1155/jonm/4829440 (PMC13770256; doi:10.1155/jonm/4829440)
Supplement: Supplementary file 2 [file JONM-2026-4829440-s002.docx]

**SANRA Checklist**

**Scale for the Quality Assessment of Narrative Review Articles (SANRA): Author Self-Assessment**

| Item | Criterion | Score (0–2) | Justification |
| --- | --- | --- | --- |
| 1 | Justification of the article's importance for the reader | 2 | The review addresses a clearly identified gap: three literatures—organizational justice, rater behavior, and measurement quality—have developed in isolation, and evidence integrating them as interdependent properties of a single assessment system remains limited. The Introduction establishes the dual administrative–developmental purposes of appraisal, the pressure for defensibility and validity, and the failure of the recent Sucipto et al. [21] systematic review to develop an integrated framework. Contemporary psychometric frameworks [3,53] are introduced to contextualize the relationship between rating quality, validity, and fairness. The review explicitly positions acute care nursing as a theoretically informative context for examining broader assessment dynamics, and it states three research questions and proposes a testable framework (three propositions) as its contribution. |
| 2 | Statement of concrete aims or formulation of questions | 2 | The Introduction states the aim explicitly: to critically synthesize and evaluate the literature on rater behavior, score information, and fairness perceptions in nursing performance appraisal, and to propose a narrower, testable framework that reframes rater behavior as an enactment condition of performance measurement. Three analytical questions structure the review: (1) What methods and approaches are used to evaluate rating quality in nursing performance appraisal? (2) What does the most direct empirical evidence establish about how rater behavior alters the informational properties of performance scores in nursing? (3) What are the consequences and impacts of rater behavior, including rater effects and fairness concerns, and what framework can integrate these mechanisms? The Abstract restates the aim under Aim(s). |
| 3 | Description of the literature search | 2 | The Methods section specifies four databases (PubMed/MEDLINE, CINAHL, PsycINFO, Scopus), three core search blocks plus one contextual block, with explicit terms, truncation (*), date limits (2000–2026), the search date (6 September 2026), and the full retrieval yield (1,247 records from databases plus 45 additional records from reference lists and citation tracking; 312 duplicates removed; 980 records screened at title and abstract level; 812 records excluded at screening; 168 full texts assessed for eligibility; 117 full texts excluded; 51 studies included in the final synthesis). Inclusion and exclusion criteria are explicitly stated. The rationale for including non-nursing literature is provided. The two-dimensional evidence classification (relevance × evidence characteristics) is described. The conceptual synthesis and framework development process is described, including the distinction between empirically supported relationships and theoretically proposed relationships. Reporting quality guided by SANRA, applied as an author self-assessment, is described in the Methods. The decision not to run a sensitivity search without the contextual filter is justified in the Methods and acknowledged as a limitation. Screening agreement (94%; 184/196 records) and verification of data extraction for a 25% sample are reported. |
| 4 | Referencing | 2 | The manuscript cites 53 references, including seminal theoretical works (Colquitt [8]; Colquitt et al. [9]; Erdogan [5]; Murphy & Cleveland [1]; Levy & Williams [4]; Messick [2]; Kane [3]; AERA et al. [53]), recent empirical studies (Polat et al. [29]; Rabenu & Shertzer [22]; Kuntarti & Rahmayanto [37]; Dexter et al. [15]; Paramesh et al. [13]), methodological standards (Mokkink et al. [46,47]; Terwee et al. [34,51]; Sijtsma & Pfadt [30]; Gagnier et al. [35]), and international evidence from Australia, Bangladesh, Canada, Chile, China, Egypt, Germany, Ghana, India, Indonesia, Israel, Japan, Jordan, the Netherlands, Nigeria, Norway, Portugal, Saudi Arabia, South Korea, Spain, Turkey, and the United States. All 53 references are cited in the main text; entries [41–53] are cited where they inform the framework and are listed in Table 4. Reference details were verified against publisher or repository records. References are formatted with full article titles and DOIs where available. |
| 5 | Scientific reasoning | 2 | The manuscript develops a coherent, evidence-based argument that rater behavior is an enactment condition of performance measurement. The Rater-Enacted Assessment System Model is supported link by link with citations, and the model explicitly distinguishes empirically supported relationships (Proposition 1) from theoretically proposed relationships requiring direct testing (Propositions 2 and 3). The authors distinguish nursing-specific evidence from transferable organizational psychology and assessment science evidence, and they explicitly identify propositions requiring empirical investigation. The Discussion converts the model's propositions into a testable research agenda. |
| 6 | Presentation of endpoint data | 2 | Table 1 presents the evidence basis for each proposed relationship, specifying evidence strength, key sources, what is established, and what is not established. Table 2 presents the architecture of the 51 included sources. Table 3 summarizes the approaches used to evaluate rating quality (Research Question 1). Table 4 presents a summary of included studies with author, year, country, design, sample, setting, principal findings, and relevance to the framework. Figure 1 depicts the study selection flow diagram with complete arithmetic reconciliation. Figure 2 depicts the conceptual model with a solid arrow only for the empirically supported pathway (rating behavior to observed score distribution; Proposition 1) and dashed arrows for all other pathways, which are theoretically proposed or only indirectly supported. Table 5 presents the three propositions with evidence status, key evidence, boundary conditions, and required tests. Tables 1–5 and Figures 1 and 2 are each cited in the main text. The Results section provides a structured presentation of findings organized by the three research questions. |
| **Total** |  | 12/12 |  |

***Rating scale:*** *0 = low standard; 1 = moderate standard; 2 = high standard.*

***Note:*** *SANRA was applied by the authors as a self-assessment of reporting quality [24,25]. It is not an independent or external rating, and the scores should be read as an indication of reporting transparency rather than of the validity of the synthesis.*
