# Supplementary material for: Rater Behavior as an Enactment Condition in Nursing Performance Appraisal: A Critical Review
Source: J Nurs Manag. 2026 Nov 15;2026:4829440. doi: 10.1155/jonm/4829440 (PMC13770256; doi:10.1155/jonm/4829440)
Supplement: Supplementary file 3 [file JONM-2026-4829440-s003.docx]

**Supplementary File 2. Complete Database Search Strings**

| Database | Search String |
| --- | --- |
| **PubMed/MEDLINE** | ("performance appraisal"[MeSH] OR "performance evaluat*"[tiab] OR "performance assess*"[tiab] OR "performance review*"[tiab] OR "employee evaluat*"[tiab]) AND ("nurse"[MeSH] OR "nurs*"[tiab] OR "nurs* manager*"[tiab] OR "staff nurs*"[tiab]) AND ("fairness"[tiab] OR "justice"[tiab] OR "rater bias*"[tiab] OR "rater effect*"[tiab] OR "lenien*"[tiab] OR "severity"[tiab] OR "halo"[tiab] OR "central tendenc*"[tiab] OR "rater motivation"[tiab] OR "appraisal motivation"[tiab] OR "rating accuracy"[tiab] OR "reliab*"[tiab] OR "validit*"[tiab] OR "measurement error*"[tiab] OR "psychometric*"[tiab]) AND ("acute care"[tiab] OR "hospital*"[tiab]) AND ("2000"[Date - Publication] : "2026"[Date - Publication]) |
| **CINAHL** | ((MH "Performance Appraisal") OR (TI "performance evaluat*" OR AB "performance evaluat*") OR (TI "performance assess*" OR AB "performance assess*") OR (TI "performance review*" OR AB "performance review*") OR (TI "employee evaluat*" OR AB "employee evaluat*")) AND ((MH "Nurses") OR (TI nurs* OR AB nurs*) OR (TI "nurs* manager*" OR AB "nurs* manager*") OR (TI "staff nurs*" OR AB "staff nurs*")) AND ((TI fairness OR AB fairness) OR (TI justice OR AB justice) OR (TI "rater bias*" OR AB "rater bias*") OR (TI "rater effect*" OR AB "rater effect*") OR (TI lenien* OR AB lenien*) OR (TI severity OR AB severity) OR (TI halo OR AB halo) OR (TI "central tendenc*" OR AB "central tendenc*") OR (TI "rater motivation" OR AB "rater motivation") OR (TI "appraisal motivation" OR AB "appraisal motivation") OR (TI "rating accuracy" OR AB "rating accuracy") OR (TI reliab* OR AB reliab*) OR (TI validit* OR AB validit*) OR (TI "measurement error*" OR AB "measurement error*") OR (TI psychometric* OR AB psychometric*)) AND ((TI "acute care" OR AB "acute care") OR (TI hospital* OR AB hospital*)) AND DT 20000101-20261231 |
| **PsycINFO** | ((DE "Performance Evaluation") OR (TI "performance apprais*" OR AB "performance apprais*") OR (TI "performance evaluat*" OR AB "performance evaluat*") OR (TI "performance assess*" OR AB "performance assess*") OR (TI "performance review*" OR AB "performance review*")) AND ((DE "Nurses") OR (TI nurs* OR AB nurs*) OR (TI "nurs* manager*" OR AB "nurs* manager*") OR (TI "staff nurs*" OR AB "staff nurs*")) AND ((DE "Justice") OR (TI fairness OR AB fairness) OR (TI justice OR AB justice) OR (TI "rater bias*" OR AB "rater bias*") OR (TI "rater effect*" OR AB "rater effect*") OR (TI lenien* OR AB lenien*) OR (TI severity OR AB severity) OR (TI halo OR AB halo) OR (TI "central tendenc*" OR AB "central tendenc*") OR (TI "rater motivation" OR AB "rater motivation") OR (TI "appraisal motivation" OR AB "appraisal motivation") OR (TI "rating accuracy" OR AB "rating accuracy") OR (TI reliab* OR AB reliab*) OR (TI validit* OR AB validit*) OR (TI "measurement error*" OR AB "measurement error*") OR (TI psychometric* OR AB psychometric*)) AND PY 2000-2026 |
| **Scopus** | TITLE-ABS-KEY("performance apprais*" OR "performance evaluat*" OR "performance assess*" OR "performance review*") AND TITLE-ABS-KEY("nurs*" OR "nurs* manager*" OR "staff nurs*") AND TITLE-ABS-KEY("fairness" OR "justice" OR "rater bias*" OR "rater effect*" OR "lenien*" OR "severity" OR "halo" OR "central tendenc*" OR "rater motivation" OR "appraisal motivation" OR "rating accuracy" OR "reliab*" OR "validit*" OR "measurement error*" OR "psychometric*") AND TITLE-ABS-KEY("acute care" OR "hospital*") AND PUBYEAR > 1999 AND PUBYEAR < 2027 |

***Note:*** *The strings for PubMed/MEDLINE, CINAHL, and Scopus include a fourth, contextual block ("acute care" OR "hospital"); the PsycINFO string does not. The term "employee evaluation" was searched in PubMed/MEDLINE and CINAHL only. A sensitivity search without the contextual block was not undertaken (see Methods and Limitations). An asterisk (*) denotes truncation (e.g., nurs* retrieves nurse, nurses, and nursing). Date limits of 2000–2026 were applied in all four databases and are shown at the end of each string (PubMed: Date - Publication; CINAHL: DT; PsycINFO: PY; Scopus: PUBYEAR). The initial search strategy applied acute-care and hospital filters in several databases. To address the potential restrictiveness of this strategy, reference list checking and citation tracking were conducted for seminal works on rater bias, organizational justice, and nursing performance measurement. This supplementary approach identified 45 additional records, including studies addressing rater behavior and performance appraisal in organizational settings beyond acute care. The supplementary search methods are described in the Methods section.*
